# Supplementary material for: The interactome of intact mitochondria by cross-linking mass spectrometry provides evidence for coexisting respiratory supercomplexes
Source: Mol Cell Proteomics. 2017 Dec 8;17(2):216–32. doi: 10.1074/mcp.RA117.000470 (PMC5795388; doi:10.1074/mcp.RA117.000470)
Supplement: Supplemental Data [file supp_RA117.000470_133922_0_supp_23978_fzffwf.zip › spectra_annotation/mito_spectra_annotation/15-1-9-1-10-1.pdf]

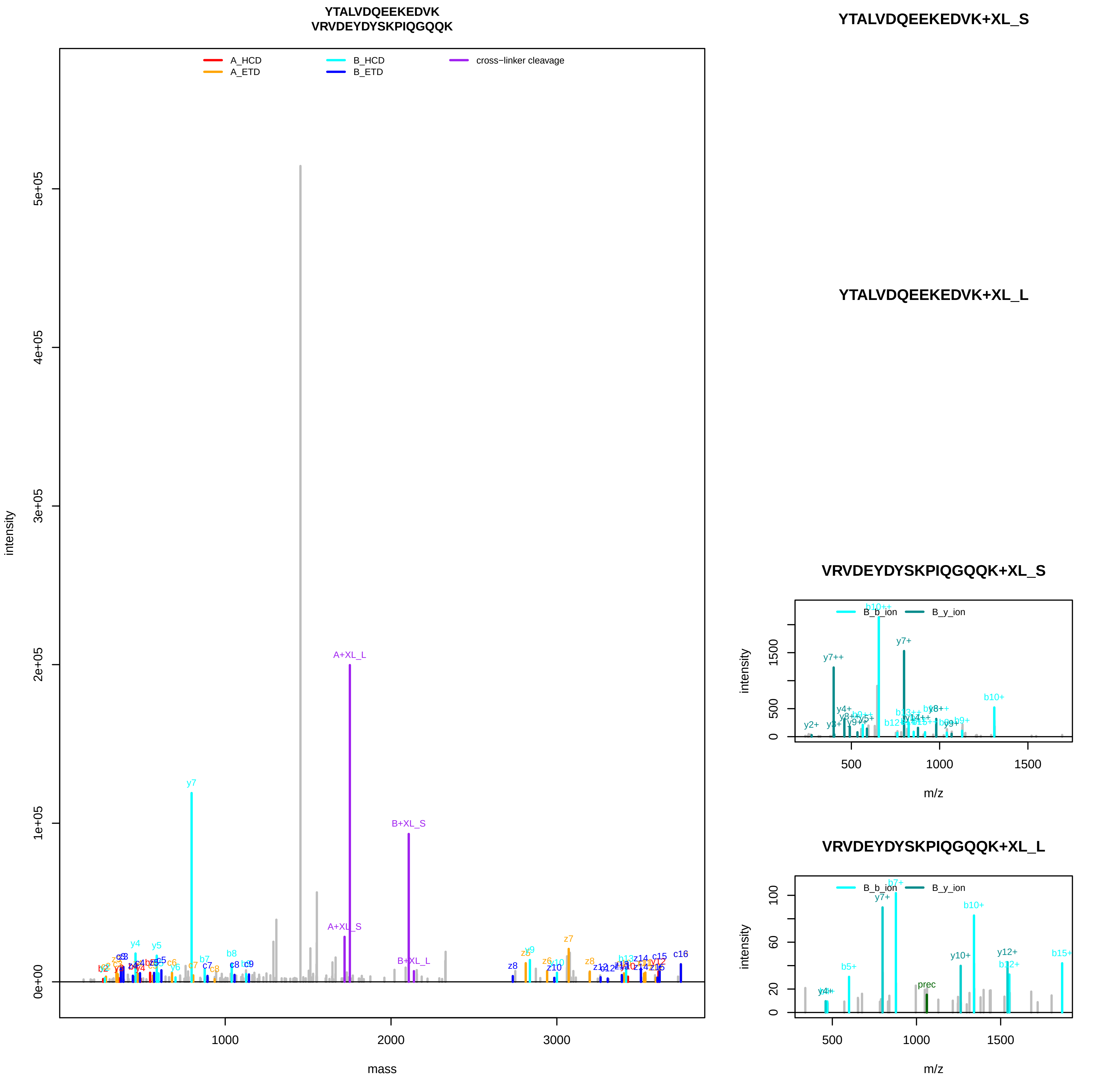

YTALVDQEEKEDVK
VRVDEYDYSKPIQGQQK
A_HCD
A_ETD
B_HCD
B_ETD
cross−linker cleavage
A+XL_L
A+XL_S
B+XL_S
B+XL_L
y7
intensity
mass
0e+00
1e+05
2e+05
3e+05
4e+05
5e+05
1000
2000
3000
YTALVDQEEKEDVK+XL_S
YTALVDQEEKEDVK+XL_L
VRVDEYDYSKPIQGQQK+XL_S
B_b_ion
B_y_ion
b10++
y7+
y7++
b10+
intensity
m/z
0
500
1500
500
1000
1500
VRVDEYDYSKPIQGQQK+XL_L
B_b_ion
B_y_ion
b7+
y7+
b10+
b5+
y10+
y12+
b12+
b15+
y4+
prec
intensity
m/z
0
20
60
100
500
1000
1500
